# Austrian economics without extreme apriorism: construing the fundamental axiom of praxeology as analytic

**DOI:** 10.1007/s11229-019-02150-8

**Published:** 2019-03-12

**Authors:** Alexander Linsbichler

**Affiliations:** DK The Sciences in Context and Department of Philosophy, University of Vienna, Althanstraße 14, UZA II, Room 2H353, 1090 Vienna, Austria

**Keywords:** Analyticity, Apriorism, Austrian School of economics, Praxeology, Conventionalism, Imre Lakatos, Intuition, Ludwig Mises

## Abstract

Current debates between behavioural and orthodox economists indicate that the role and epistemological status of first principles is a particularly pressing problem in economics. As an alleged paragon of extreme apriorism, the methodology of Austrian economics in Mises’ tradition is often dismissed as untenable in the light of modern philosophy. In particular, the defence of the so-called fundamental axiom of praxeology—“Man acts.”—by means of pure intuition is almost unanimously rejected. However, in recently resurfacing debates, the extremeness of Mises’ epistemological position has been called into question. Rather than directly engaging in these exegetical discussions, this paper aims to substantiate the possibility and plausibility of conventionalist defences of praxeology per se. The proposed shift includes settling for an analytic fundamental axiom and acknowledging the prima facie tenability of other research programs than praxeology. Since conventionalist praxeology is only moderately aprioristic, mainstream economists and philosophers might be more likely to engage in fruitful discussions with those Austrian scholars who elaborate pragmatic arguments for praxeology instead of invoking pure intuition.

## Introduction: First principles in (Austrian) economics

In each discipline of empirical science, methodologists face a tension between empirism and aprioristic foundationalism. On the one hand, empirical sciences require falsifiability or at least some weaker version of contact to “the observable world”. On the other hand, any inquiry must start from some first principles which are not in fact doubted and tested. The role of these first principles is particularly eminent if they serve as axioms of pure deductive systems. (Blaug 1985, pp. 697–698; Hoover 2017; di Iorio 2008).

As one salient and current example, empirical findings of behavioural economics have permeated the protective belt of some axioms of orthodox (descriptive) rational choice theory. In these ongoing debates, orthodox economics is accused of ignoring empirical findings, violating fallibilism, and adhering to an untenable foundationalist apriorism.^1^ Ironically enough, orthodox economics itself blames Austrian economics for “the exaggerated claims that used to be made in economics for the power of deduction and a priori reasoning” (Samuelson 1964, p. 736). Particular criticism is directed at praxeology—the “cranky and idiosyncratic” (Blaug 2006, p. 81) methodology explicated by Mises: “Mises’ method is perhaps the most anti-positivist and anti-empiricist approach to social science ever stated.” (Milonakis and Fine 2009, p. 259)

According to a widespread conviction, at least the subgroup of Austrian economists who work in the praxeological tradition depends on an extreme form of apriorism that is untenable in the light of modern philosophy of science. Consequently, “throughout the history of the last century of economic discourse, [extreme apriorism has] been used time and again as a too-simple excuse to reject otherwise fine theoretical lessons developed by Austrian-School economists” (Scheall 2017a, p. 240). Along similar lines, Zanotti and Cachanosky motivated their reconstruction of Mises’ epistemological position as only moderately aprioristic by arguing that without extreme apriorism

[T]he Austrian parallel economic world would not have lost its relevance and more gains from trade could have taken place between the two paradigms. […] Especially after the Austrian revival in 1974, communication between Austrians and non-Austrians could have been much more productive. […The praxeological] economic approach would not have been considered too idiosyncratic to have been taken seriously. (Zanotti and Cachanosky 2015, p. 131)

The recent challenge whether the apriorism championed by Mises is actually extreme triggered considerable criticism (see Gordon 2014; Cachanosky 2014; Zanotti and Cachanosky 2015, 2017; Scheall 2017a). In comparison to the exegetical problem about Mises’ epistemological position, the paper at hand is primarily concerned with substantiating the possibility and plausibility of a defence of praxeology that is only moderately aprioristic—no matter whether it concurs with Mises’ intentions or not. Given such a more tenable justification of praxeology, we hope that proponents of Austrian economics who are interested in constructive interaction and scientific progress will subsequently find more like-minded colleagues within the mainstream and within other heterodox schools of social scientific thought.

Section 2 introduces to praxeology, following its standard interpretation: We sketch the idea and the content of praxeology (Sect. 2.1), we show that praxeology is not extremely aprioristic with respect to the scope of its apriorism (Sect. 2.2), raise problems of justifying praxeology (Sect. 2.3), and relate our paper to prominent Austrian economists who dispense with praxeology (Sect. 2.4). Section 3 outlines our new, conventionalist proposal for justifying praxeology: We expound some general features of conventionalism (Sect. 3.1), argue why conventionalism ideally suits the content of praxeology (Sect. 3.2), discuss previous Austrian steps towards conventionalism (Sect. 3.3), and rectify some common misattributions to conventionalism (Sect. 3.4). Section 4 adumbrates the double-edged prospects of praxeology if it adopted a more modest epistemological position.

## Mises’ theory of human action: praxeology

### The idea of praxeology and the fundamental axiom

Depending on context, “praxeology” denotes a specific methodology for the social sciences or a theory developed employing this methodology. Praxeology is the distinguishing feature of the branch of Austrian economics maintaining to work in Mises’ tradition. That being said, praxeology, its proper role, and its justification are contested even within the Austrian School. While many Austrian economists may not identify their methodological approach with praxeology or Mises, almost all of them share at least some moderate variety of apriorism and sometimes suffer from guilt by supposed association with praxeology’s extreme apriorism. Section 2.4 outlines some congruities and divergencies between Mises as a representative of praxeology on the one hand and Hayek, Schütz, and Lachmann on the other.

The basic idea of praxeology as presented by Mises takes a marked stand between the poles of empirism and aprioristic foundationalism. Aiming “to establish the logical legitimacy of the science that has for its object the universally valid laws of human action” (Mises 2003 (1933), p. lxxvii), Mises criticized several other approaches to the social sciences for their reliance on empirism. Among the rejected epistemological positions are the inductivism of the German Younger Historical School, behaviourism, what Mises held to be Neurath’s physicalism, Spann’s intuitive universalism, and Popper’s hypotheticism.^2^ According to Mises, an irrefutable, true starting point of social scientific theory is required:

Praxeology- and consequently economics too - is a deductive system. It draws its strength from the starting point of its deductions […]. No economic theorem can be considered sound that is not solidly fastened upon this foundation by an irrefutable chain of reasoning. A statement proclaimed without such a connection is arbitrary and floats in midair. (Mises 1998 (1949), p. 68)

Once an a priori true starting point is established somehow, truth-preserving deduction allows for the advancement of an a priori true social scientific theory which consists of all the logical consequences of the starting point. Empirical findings can neither confirm nor falsify, but only illustrate the derived praxeological laws.^3^ Therefore, the development of economic theory can be conducted in an armchair, just like logic and mathematics, says Mises (2012(1962), p. 78).^4^

Fortunately for praxeologists, Mises believed he actually found and satisfactorily justified such a desired a priori true starting point: “Man acts.” Following Rothbard, we will refer to this proposition as the fundamental axiom of praxeology. Mises somewhat elaborates on its implicit content^5^:

The proposition: Man acts, is tantamount to the proposition: Man is eager to substitute a state of affairs that suits him better for a state of affairs that suits him less. In order to achieve this, he must employ suitable means. (Mises 2005 (1957), p. 179)

Although this paper mainly addresses the epistemological status of the fundamental axiom, a few clarifications of its intended content might be in place: For praxeologists, action is purposeful behaviour that aims for the removal of subjective uneasiness. By contrast, a reflex to a stimulus might often be considered no action, depending on the possibility to stop the reflex.^6^ Moreover, the usage of the fundamental axiom strongly indicates that it should be explicated as: “Man acts and only man acts.” Specifically, praxeologists usually follow Mises in ascribing goals, knowledge, preferences, or intentions to individual human beings, but not to social collectives, animals, or inanimate objects like swamps or door handles. In the Austrian School, acting individuals are thought of as active and potentially creative problem solvers, who possess incomplete and possibly inaccurate knowledge. They can err in their interpretations and choices.

### The extent of a priori knowledge according to Mises

Mises’ epistemological position and the approach of the praxeological branch of the Austrian School in general have been accused of extreme apriorism. To be sure, if apriorism was a sound position, its extremeness would be an asset rather than a problem. Of course, every scientist would rejoice over knowledge about empirical facts and regularities that is far-reaching, certain, and can be extended without leaving the armchair in office. The desirability and soundness of versions of extreme apriorism has indeed been defended by some Austrian economists. (Rothbard 1997b (1957), pp. 100–108; Hoppe 1995; Smith 1996)

While we concur with the overwhelming majority of economists and philosophers of science who are at least highly sceptical about the tenability of extreme apriorism, the aim of this paper is neither to present another criticism of extreme apriorism nor to defend conventionalism against criticisms put forward by Popper, Quine,^7^ and others. Like any epistemological position, conventionalism of course entails specific difficulties, such as the necessity of implicit definitions. Our more modest objective is to demonstrate that widely deprecated forms of extreme apriorism are not necessary to justify the adoption of the fundamental axiom and the implementation of praxeological methodology. If praxeology does not depend on extreme apriorism, ideas of the Austrian School are less likely to be repudiated beforehand on epistemological grounds.

What is more, our proposed conventionalist defence of praxeology allows praxeologists to keep the content of the fundamental axiom and of deductively inferred theory unaltered. To put it straight, we retain the content of Mises’ praxeology, but we advance a new justification. Accordingly, for the most part the paper addresses the recently resurfaced exegetical debate on Mises (Gordon 2014; Cachanosky 2014; Zanotti and Cachanosky 2015, 2017; Scheall 2017a) indirectly at most. We do not ask how extreme Mises’ apriorism really was. Instead, Sect. 3.2 proposes a virtually unexplored, conventionalist defence of praxeology. The extremeness of our conventionalist praxeology and its overall expediency, should in any case be judged on its own merits.

Following Scheall (2017a), we assess the extremeness of an aprioristic position along three different dimensions: (i) the *extent* of a priori knowledge, (ii) the *kind of justification* for a priori knowledge,^8^ (iii) the purported *certainty* of a priori knowledge in relation to the certainty of knowledge in other scientific disciplines.

As to the extent dimension of extremeness (i), solely the fundamental axiom ‘Man acts.’ and its logical consequences are a priori in Mises’ praxeology. Postulating that only individuals have ends and attain means to attain them, is certainly a very narrow basis for social scientific theorizing. For instance, the usual assumptions of contemporary neo-classical models exceed the content of the Austrian fundamental axiom by far. Furthermore, compared to other proponents of apriorism and deductivism in the history of economic thought, praxeology proposes a very narrow apriorism.^9^

Over and above the thinness of the fundamental axiom, further evidence for the moderate character of the extent of Mises’ apriorism can be found in the indispensability of fallible, empirical aspects of the sciences of human action: auxiliary axioms and thymology.

Firstly, alongside the fundamental axiom, numerous definitions and auxiliary axioms, such as the disutility of labour, allow for the extension of praxeological theory.^10^ In scientific contexts it is predominantly recognized that the auxiliary axioms are not required to be a priori by Mises and that they consequently do not enhance the extremeness of Austrian apriorism. In public discourse though, self-proclaimed Miseseans sometimes arrogate a priori truth and alleged apodictic certainty for praxeological theorems^11^ that in fact rely on auxiliary axioms, and even for normative claims in ethics and politics. These imprecisions together with an extremely aprioristic epistemological position may partly account for the dogmatic style and attitude sometimes displayed in such debates in broader contexts.^12^

Secondly, due to the focus on a priori praxeology in discussions of Mises’ methodology, the second branch of his sciences of human action, thymology, tends to receive little attention only. Thymology aims at grasping people’s value judgements, wishes, desires, preferences, and meaning assignments, but without using methods of the natural science. Instead, a method of historical understanding (Verstehen) is invoked. Although results of this thymological Verstehen are hardly reliable, according to Mises only thymology is suitable for the task at all. “The Verstehen […] is the method which all historians and all other people apply in commenting upon social events of the past and in forecasting future events.” (Mises 1990 (1944), p. 24) Despite the limitations of thymology, Mises deems it an integral part of Austrian economics: “Both [praxeological] theory and [thymological] history are equally legitimate, and both are equally indispensable.” (2003 (1933), lxxviii) Specifically, explanations and predictions in the social sciences consist of at least one praxeological theorem, at least one sentence obtained thymologically describing boundary conditions, and potentially sentences from logic, mathematics, or the natural sciences.^13^

Providing boundary conditions for explanations and predictions by no means exhausts the function of thymology. Another crucial utilization is relating praxeological concepts and sentences to the external world. Considering “exchange” as a comparatively simple concept, Long elaborates on a Wittgensteinian motif in order to substantiate the difficulties in establishing whether observed behaviour is exchange or, say, a religious practice. (Long 2013, pp. 23–32, 101–104) Yet even more fundamentally, if a scientist observes behaviour, praxeology tells her that if this behaviour was an action, then the actor preferred something to something else. However, this a priori knowledge is almost useless without thymology. Whether a specific behaviour was an action at all, and if so, what was preferred to what, are aposteriori hypotheses.^14^

As a drastic example, consider Hausman’s (2011, pp. 27–28) Romeo. Evidently only a very bad thymologist would maintain that Romeo drank poison because he preferred dying to eloping with Juliet. Contrary to (unsophisticated versions of) a revealed preference approach, the demonstrated preference approach, which often accompanies praxeology, tends to acknowledge its own hypothetical and thymological character slightly more explicitly.^15^ This includes, as Austrian economists would insist, a reconstruction of the (often incomplete or erroneous) knowledge of Romeo.

Contradicting Mises, it has been suggested and rudimentarily implemented to amend thymological Verstehen with other empirical means to grasp intentions, meaning assignments, and value judgements. (Rizzo 1978; Pavlik 2006; Linsbichler 2017) In any case, apriorism remains restricted to the praxeological compartment of the social sciences. Neither Mises nor most of his contemporary successors are proponents of extreme apriorism regarding the *extent* dimension. We will examine the two remaining dimensions of extremeness, *kind of justification* (ii) and *certainty* (iii), too. These dimensions do not depend on the content of the fundamental axiom, but on its justification. Thus we will return to them at later points.

### Different interpretations of praxeology

While it is evident that “[p]raxeology is a priori” (Mises 2012 (1962), p. 44), even well-meaning followers of Mises admit that his use of the term is idiosyncratic and has caused confusion (Rothbard 2007 (1985), p. xv). Thus, even Austrian economists who consider themselves in the succession of Mises have disagreed regarding the interpretation of “some of his more cryptic statements” (Kirzner 2001, p. 195), including the epistemological status of the fundamental axiom. Schütz even recommends dropping the confusing term “a priori” altogether. (Kurrild-Klitgaard 2001, pp. 127–128)

However, most interpreters inside and outside the Austrian School do agree that the justification for the fundamental axiom rests on intuition, pure intuition, pure reason, or rationalist introspection.^16^ Particularly, Mises’ is often portrayed as a Kantian or Neo-Kantian, who tried to establish the fundamental axiom as a synthetic judgement a priori. (Otter 2010; Milford 1992; Hoppe 1995; Prychitko 1998; Radnitzky 1995; Rothbard 1997d (1973), p. 32, 1997b (1957), p. 105) All those faculties have in common that experience of the outside world has no part in them and that they allegedly justify an axiom that is necessarily true and highly certain in comparison to the tentative hypotheses of the natural sciences and of thymology. The standard reading of the justification of the truth of praxeology therefore qualifies as extreme apriorism with regard to the *kind of justification* (ii) dimension and the *certainty* (iii) dimension. (Scheall 2017a)

Among contemporary philosophers and economists, the idea of synthetic judgements a priori and more generally speaking any form of extreme foundationalism based on intuition is hardly rated as a tenable position. Accordingly, the extreme apriorism of the standard account of praxeology has been harshly criticized. (Blaug 2006; Samuelson 1964; Hutchison 1981; Radnitzky 1995) Even benevolent commentators tend to be sceptical or ascertain a need for clarification (White 2003 (1977); Zilian 1990; Hayek 1978a; Caldwell 1984; Nozick 1977). Nevertheless, extremely aprioristic justifications of praxeology by Mises, Rothbard, and others have hitherto remained dominant. Before we present our conventionalist alternative in Sect. 3, three other non-standard interpretations of the fundamental axiom should be mentioned and briefly discussed in turn: empirical statements based on observation, methodological rule, and genetic or psychological a priori.

Mises once puzzled Kirzner by a remark in private conversation to the effect that the fundamental axiom can be justified by observation. (Kirzner 2001, pp. 88–89) Albeit, numerous sections in Mises’ own writings as well as in the digressions of other Austrian scholars controvert this idea. A fallible observational statement is not eligible as a fundamental axiom of praxeology.

Tokumaru (2009) suggests interpreting the fundamental axiom as a methodological rule or a methodological principle. Strictly speaking, a methodological rule is not an axiom or a theorem of a theory and hence has no truth value, but normatively determines how to apply a theory and how to adapt truth values of parts of a theory. Therefore, the interpretation as a methodological rule in a strict sense does not yield an appropriate epistemological role for the fundamental axiom of praxeology.^17^

Finally, some formulations in Mises’ methodological writings, especially in (Mises 2012), seem to imply that the fundamental axiom is a genetic or psychological a priori.^18^ This concept refers to bodily or mental equipment that invariably conditions and shapes human experience and expectations. Thus, genetic or psychological a priori must not be confused with a priori truth. (Popper 2010 (1930–1933), pp. 22–166) Since it is the latter which praxeologists proclaim for the fundamental axiom,^19^ the interpretation of “Man acts.” as a genetic or psychological a priori is deficient.^20^ The desired a priori truth of the fundamental axiom is obtained by the conventionalist justification in Sect. 3.2, namely as analytic truth.

### Austrian economics without praxeology

#### Hayek and the a priori: fallible and too limited for praxeology

There were Austrian economists before Mises’ explication of praxeology; and many Austrian economists today do not champion praxeology as outlined in Sect. 2.1.^21^ However, we have been almost exclusively concerned with the praxeological branch of Austrian economics so far. We conclude Sect. 2 with an outlook on the work of Hayek, Schütz, and Lachmann.

The portrayal of their positions will inevitably remain somewhat cursory due to open interpretational problems regarding their conceptions of the a priori and their relationship to praxeology. The secondary literature on these problems contains vehement exegetical disputes and the acknowledgement of research lacunae. For instance, the correct interpretation of the development of Hayek’s epistemological position is almost as contested as Mises’. Consequently, what Hayek thought about praxeology and about Mises’ conception of the a priori is highly controversial. Scheall (2015a, b), although he has “skin in the game”, presents a balanced and insightful overview of the history of the debates regarding Hayek’s epistemological position and his purported turns. Contrary to Hutchison’s still influential received view, Scheall opines that Hayek was a fallibilist throughout his life^22^ and concludes that “Hayek and Mises defended mutually inconsistent notions of a priori knowledge such that, even if Hayek had accepted methodological apriorism (a claim he always denied), it would have meant something different to him than it meant to Mises” (Scheall 2015b, p. 42).

In any case, the concept of a priori does not play a highlighted role in Hayek’s theorizing, much less does praxeology. Particularly, (Hayek 1948 (1937)) is often portrayed as intended criticism of Mises’ praxeology and as Hayek’s ultimate departure from it.^23^ The fundamental axiom by all means does not play a central role in Hayek’s theorizing. He does not primarily aim at an encompassing theory of human action but engages in piecemeal situational analyses and pattern predictions.

Hayek’s apriorism seems to be less extreme than Mises’ with regard to all three of Scheall’s (2017a) dimensions: Hayek allows for a more eminent role of experience (kind of justification dimension ii) and for fallibilism (certainty dimension iii). Moreover, Hayek’s pure logic of choice (1948 (1937)) restricts a priori reasoning from social phenomena like the market to individual actions (extent dimension i). According to him, a priori reasoning cannot be extended to social situations with several actors. As a sole basis for the far-reaching aspirations of praxeologists and many other social scientists, Hayek’s a priori is insufficient.^24^

#### A focus on thymological Verstehen: Schütz and Lachmann

Mises’ indirect and often unidentified influence on the social sciences via Schütz and his disciples Berger and Luckmann should not be underestimated. However, the exact relation between their endeavours is subject to discussions.^25^

Schütz’s interpretative sociology of the life-world (1967 (1932)) can with some crucial reservations be considered as a modification of Mises’ praxeology. Their approaches differ, among other things, in focus. Mises is fully aware that thymology and its method Verstehen are indispensable for the social sciences, but his own work focuses on a priori true praxeological theory. In comparison, Schütz concentrates on elaborating the thymological method of Verstehen and amends it with his sociology of communication. Both Mises and Schütz emphasize the continuity of Verstehen in the social sciences and Verstehen in day-to-day life. However, Schütz accentuates that interpretation is always an integral part of social scientific experience and he is also much more explicit and careful in distinguishing between two levels on which behaviour can become purposeful action by the attribution of meaning. Firstly, the actor himself can attribute meaning to his behaviour. Secondly, social scientists (and laymen in day-to-day life) can attribute meaning to the behaviour of other actors.

With regard to the a priori, Schütz carves out the structure of action as a necessary prerequisite of any application of the method of Verstehen. It seems that to be a prerequisite of thymology is the main function of the a priori in his account. Mises recognizes this function of the a priori but is not predominantly concerned with it. Furthermore, Schütz’s typifications of action indicate a drift with regard to “a priori” itself. In the tradition of Weber and collaborating with Kaufmann,^26^ Schütz constructs ideal-types and uses them as a necessary prerequisite for understanding individual action and social phenomena. This contrasts strongly with praxeology, since ideal-types of action do not aspire to apply universally to every human action with necessity.

Like Schütz, Lachmann is strongly influenced by Weber, interested in thymological interpretation (Verstehen), and aims at deploying Verstehen to classes of actions instead of individual action only. In doing so, Lachmann warns of the many pitfalls of aggregation and (like Schütz but unlike Mises in his praxeology) engages in typifications. However, these ideal–typical constructions lack the universality and necessity of a fundamental axiom of praxeology. Thus, Lachmann’s prerequisites of interpreting human action are presumably not a priori in the relevant sense.

Over and above having a different epistemological status, Lachmann’s prerequisites differ in content. Situatedness of action and incomplete knowledge of actors are two cornerstones of Austrian economics, but Lachmann takes them a step further. According to him, economics must incorporate the fact that oftentimes knowledge is lacking altogether. Particularly, the actors’ learning from past experience involves an inextricable interpretative element and, more drastically, under the prevailing conditions of radical uncertainty, even if actors learn anything from the past, they only learn something about the past, not about the future. This is a key factor why Lachmann ultimately arrives at different conclusions than Kirzner and Mises, regarding for instance the equilibrating tendencies of economic systems.^27^

As another partial contrast to Mises, Lachmann seems to doubt the importance of Neo-Kantianism not only for praxeology, but even for thymology: “But is there really a lot that we can learn from Rickert and Windelband today?” (Lachmann 1956; translated by the author) In response, Mises dignified Rickert’s and Windelband’s contribution to thymology. With regards to praxeology however, Mises considered them as ignorant as Weber, Bergson, and Husserl, who is renowned as Schütz’s principal influence exceeding Weber and Mises. According to the latter, we can learn a lot from Husserl, if we seek to become wiser, but nothing in order to establish the truth of a fundamental axiom of praxeology. (Mises 1956)

This paper primarily addresses the allegedly most extreme form of apriorism in the Austrian School, praxeology. If the aprioristic parts of the theories of Hayek and other Austrian economists are indeed more moderate, then a transfer of the proposal in Sect. 3 from praxeology to, say Hayek’s pure logic of choice or Schütz’s interpretative sociology of the life-world, should pose little difficulties.^28^

## A conventionalist justification for praxeology

### Conventionalism: How some philosophers of science keep all swans white

In Sect. 3.1 we provide a broad characterization of conventionalism. More specific or even slightly divergent definitions exist, but our characterization is in line with most historical and contemporary versions of conventionalism.^29^ Subsequently, Sect. 3.2 argues that praxeology qualifies for an uncontroversial version of conventionalism and could avoid accusations of extreme apriorism. We eventually discuss previous traces of analyticity and conventionalism in the writings of Austrian economists (Sect. 3.3) and finally tackle some potential misunderstandings and criticisms of conventionalism (Sect. 3.4).

We can characterize a conventionalist epistemological position in general as an approach to dealing with a theory in the face of new evidence. A conventionalist fixes the truth value of at least one sentence of her theory, at least for the time being. For this conventional part of a theory, neither observation nor intuition are a critical standard by which to falsify, verify, confirm, disconfirm, or corroborate it. There are two main immunizing strategies, which we will illustrate by rehashing the philosopher’s standard example “All swans are white.”. Keeping some reservations in mind, economists can substitute “All demand curves are downward sloping.”.^30^

Consider the law “For all x: If x is a swan [where “swan” is defined as having the properties P_1_, P_2_, and P_3_], then x is white.” and let it be fixed as true by convention. This decision might for instance be justified by myriads of observed white swans and the importance of this law for further theoretical development. Now reports of a black swan reach the conventionalistically minded ornithologist. A first immunizing strategy is to simply discard these reports. The sentences which describe black swans and thereby contradict the convention might be based on optical illusions or political bias. In the case of praxeology, potential findings that would contradict theorems of praxeology necessarily involve statements about the preferences, knowledge, goals, or intentions of individuals. According to Mises, such statements can only rest on thymology and are therefore highly uncertain. Therefore, thymological statements are prime candidates for being discarded in case of doubt. In general though, the first immunizing strategy facilitates dogmatic tendencies.

In comparison, the second immunizing strategy is far less problematic, partly due to its higher transparency. Thus, we will focus on it in the remainder of the paper. In a conventionalistic theory, not all terms are fixed in their meaning by applicative definitions.^31^ Instead, the meaning of at least one term is fixed only insofar that it must refer to concepts, phenomena, or objects, such that the sentence in question stays true. Oftentimes, conventionalism seeks simple laws of nature, perhaps at the expense of very complex and changeable applicative definitions for the terms of the theory.

Now let us apply the second immunizing strategy to our swan example, i.e. we keep the (syntax of the) law and its truth-value, but we adapt its semantics. In this way, we can accept the report that a black object with the properties P_1_, P_2_, and P_3_ exists and nevertheless we keep the sentence “For all x: If x is a swan [where “swan” was heretofore defined as having the properties P_1_, P_2_, and P_3_], then x is white.” true. The trick is to change the meaning of “swan”, for instance by requiring an additional property P_4_ which the newly discovered black object does not have. A trivial and often unhelpful choice for P_4_ would be “white”. In this case, our updated and now rather boring law would read “For all x: If x is a swan [defined as being white and having the properties P_1_, P_2_, and P_3_], x is white.” Note however, that conventionalism by no means requires all its laws to be trivial.

Howbeit, the truth value of at least one sentence of a conventionalist theory is not directly determined by observation or intuition, but by a methodological *decision*. A sentence which is true by convention is kept true by adapting the meaning of the terms involved accordingly if need be. Two necessary features of conventionalism therefore are in any case: *(I) The conventions could in principle have been chosen differently, i.e. alternative theories or research programs are possible. (II) The conventions are not justified by observation or intuition, but by pragmatic arguments for the superior expediency of the resulting theory or research program.*^32^

This paper not only argues that praxeology can be defended conventionalistically ((I) and (II)). Moreover, we claim that praxeology qualifies for one of the least controversial versions of conventionalism. In order to do so, a defence of the fundamental axiom must additionally meet a third condition: *(III) Conventions are restricted to sentences that do not exclude any potentially observable*^33^*states of affairs.*

### Construing an “ultra-refined grammar” for Austrian economics

We propose a shift from a synthetic fundamental axiom to an analytic one, analogous to the elimination of judgements which are allegedly synthetic a priori from the disciplines of logic, mathematics, and of parts of physics. In these disciplines, logical empiricism showed how first principles can be justified as being linguistic conventions (or synthetic statements a posteriori) instead of resulting from dubious intuitions. This paper proposes an analogous shift for praxeology. If the fundamental axiom of praxeology is true by virtue of meaning, it is analytic.

Indeed, Mises stresses the epistemologically identical character of praxeology and its “a priori sibling sciences logic and mathematics” (1940, p. 63).^34^ No empirical findings falsify, verify, or confirm mathematical theorems. The same goes for other analytic and thus true statements like “All husbands are married.” and “[C]eteris paribus, an increase in the quantity of money leads to a decrease in the purchasing power of the monetary unit” (Mises 2003 (1933), p. 128).^35^

A hitherto unappreciated but crucial similarity between logic, mathematics, parts of physics, and praxeology is that mathematical terms do not refer to observable objects, and that praxeology on closer examination has no obvious empirical content either. The fundamental axiom in effect only stipulates that certain theoretical entities exist, and certain others do not, but it does not exclude any potentially observable states of affairs.^36^ Following the fundamental axiom, most Austrian economists and many other social scientists attribute theoretical, unobservable entities like preferences, interests, goals, meaning assignments, value judgements, and subjective knowledge to individual men, but not to planets, atoms, and nations. Man acts and inanimate objects merely behave. However, there is no obvious observable difference between acting and behaving—rather the social scientist has to start with a methodological *decision*, which kind of explanations she regards satisfactory.

In order to motivate the shift toward an analytic fundamental axiom further, note that we are acquainted with theoretical entities like goals, means, and preferences from our everyday lifeworld. Nearly all laymen use a language containing such theoretical, i.e. non-observable, terms in explanations of what humans do. Although such familiarity is no guarantee for scientific progress as physics indicates, we may welcome that Mises’ depiction of “the fundamental law of action” is true by virtue of the usual meanings of its terms in everyday language: By virtue of meaning it is true that what is subjectively considered more important is, ceteris paribus, preferred to what is subjectively considered less important. (Mises 2003 (1933, pp. 87–88) Together with additional definitions, the fundamental axiom in effect (just) provides an “ultra-refined grammar” (Hutchison 1998, p. 68) for thinking and communicating about social scientific phenomena. How analyticity can be extended from the fundamental axiom to praxeological theorems is illustrated by Klein (2009): Using concepts different from neoclassical economics, the praxeological law of demand holds without any exceptions such as Giffen goods.

Analyticity of praxeology should not come as a surprise. As a matter of fact, Blaug holds that “the history of economics is certainly replete with tautological definitions and theories so formulated as to defy all efforts of falsification” (1985, p. 697). Especially the fundamental axiom has repeatedly been charged for being almost vacuous. Nelson (1992) provides a trenchant example of this, when he paraphrases Caldwell’s version of a fundamental axiom:

In fact, in plain English, Caldwell’s stated principle, *Humans have goals, and in pursuing them will respond to perceived changes in constraints,* amounts simply to this: *If someone wants something they will try to do what they think will get it for them.* It is notorious that this is suspiciously close to being a tautology. (Nelson 1992, p. 153)

Note, how the strategy of construing the fundamental axiom as analytic embraces Nelson’s charge and argues that “Man acts.” and the whole praxeological theory derived from it are true and indeed have no empirical content whatsoever. This fulfilment of condition (III) enables praxeologists and other Austrian economists to adopt a mild, less controversial form of conventionalism, in which the meaning of all observational terms can remain fixed. Applied to the case of praxeology, condition (III) boils down to the following: Since the fundamental axiom on its own does not exclude or predict any observable phenomena, it is extremely unlikely that any observation actually prompts the applicative definitions of the only observable term in the axiom, “man”, to be changed in order to keep the fundamental axiom true.^37^

Once the fundamental axiom is analytic, its conventional character—i.e. the fulfilment of conditions (I) and (II)—stands to reason. If the linguistic rules governing the analytic concepts do not serve their pragmatic purpose expediently, new conventions are to be set up.^38^

The proposal in this paper is conventionalist in an even twofold way. Firstly, the fundamental axiom decides the question which objects act by convention. This includes the landmark decisions between the alternatives behaviourism (“No objects act.”) and purposeful action (“Man and only man acts.”) as well as between strict methodological individualism and social ontologies^39^ with acting collectives. Furthermore, some fine tuning is required too, such as the determination of the difficult cases of animals and children.^40^ Adopting and explicating an analytic fundamental axiom is tantamount to the (hopefully practical and fruitful) modest proposal to talk about human behaviour using certain concepts, and to use different concepts when talking about inanimate objects.

Secondly, once it is established which objects act and which do not, many details of the concept of action allow for more than one explication.^41^ For a conventionalist there is no aspiration to ultimately settle on one allegedly unique correct meaning of action.^42^

In this section we contended that the content of Misesean praxeology is ideally suited for a conventionalist justification—which neither Mises nor his successors consistently provided. The proposed conventionalist justification evades charges of extreme apriorism as we will show in Sect. 4.

### Approaching conventionalism

#### The fundamental axiom as a Lakatosian hard core versus Rothbard’s extreme apriorism

At this point, we deem it proper to mention that some authors have questioned before whether praxeology is necessarily linked to extreme apriorism. Notably, a few scholars (Rizzo 1982; Schmid 2010; Schröder 2010; Zanotti and Cachanosky 2015, 2017) have attempted to reconstruct praxeology or Austrian economics in a proto-Lakatosian framework. Their constructions resemble our proposal and were driven by similar motivations: “The Lakatosian structure […] would have allowed Austrians to connect, and present their work, with non-Austrian economics, rather than being seen as poles apart.” (Zanotti and Cachanosky 2015, p. 132) Sect. 3.3.1 sketches the idea of these Lakatosian reconstructions as precursors to our conventionalist proposal.

Rizzo (1982) elaborates the first explicitly Lakatosian reconstruction of Austrian economics. He concisely reviews the idea of a Lakatosian research program^43^ with its hard core, protective belt, and positive heuristics in order to apply it to Austrian economics. For our present interests, the hard core is most relevant. It contains those sentences of Austrian theory which are reconstructed as a priori. Although Rizzo does not mention the term “praxeology” once, he aptly identifies the hard core of Austrian economics as one single axiom, namely “man acts or, equivalently, that he engages in purposeful behaviour” (1982, p. 57). However, when Rizzo examines a more detailed picture of the hard core, he enumerates several corollaries allegedly derivable from his single axiom. For some of these corollaries, implicit auxiliary assumptions seem to be necessary. For instance, to deduce “there is a tendency toward coordination [of individual actions]” (1982, p. 59) from the fundamental axiom presupposes that actors have some knowledge and learn at least sometimes. Apparently, Rizzo’s hard core of Austrian economics encompasses more than the fundamental axiom and possibly even excludes potentially observable states of affairs.^44^ Thereby, requirement (III) is violated and only a more controversial form of conventionalism could be developed based on Rizzo’s augmented hard core.

Anyhow, Rizzo took an auspicious first step towards Lakatosian reconstructions of Austrian economics and enables revealing comparisons between different research programs.^45^ These promising beginnings notwithstanding, Lakatosian reconstructions have hitherto hardly achieved their goal to “dispel the widely held view that Austrian methodology is eccentric and based on antiquated and incoherent logical foundations” by giving “the sense in which economic statements are nonfalsifiable or a priori” a conventionalist flavour (Rizzo 1982, p. 54). At least one systematic and one more sociological factor could have contributed to the limited success of Lakatosian reconstructions of Austrian economics. We will address these two obstructive factors in turn.

Systematically speaking, previous Lakatosian reconstructions of praxeology have failed to address the conventionalist character which Lakatosian hard cores have by definition. Can a hard core of praxeology meet requirements (I), (II), and ideally also (III) for qualifying as conventionalist? The previous failure to adequately address this crucial question justifies Scheall (2017a, p. 236–238) to criticize proto-Lakatosian reconstructions of praxeology as too far a stretch given numerous passages in which Mises and other praxeologists violate conditions (I), (II), or (III). Even Lakatos himself (1978, p. 10, p. 90) interpreted Mises as an extreme rationalist.

This, however, does not preclude the possibility of dispensing with extremely aprioristic *justification*s of praxeology in favour of conventionalism, while leaving the *content* of praxeology untouched. In Sect. 3.2, we explicitly argued that the existence and acceptance of alternatives to the hard core, to the fundamental axiom, is principally coherent with Austrian economics (I), that justifications of the fundamental axiom can be based on pragmatic considerations of expediency instead of intuitive revelation or direct empirical confirmation (II), and that an analytic reading is congenial to the notoriously vacuous fundamental axiom (III). The possibility of fulfilling the requirements (I), (II), and (III) invalidates accusations of necessarily extreme apriorism which praxeology has often faced. Given this rebuttal of systematic objections to a conventionalist justification of praxeology, the *content* of praxeology as outlined in Sect. 2 could subsequently be reconstructed in a Lakatosian framework.

On a more sociological note, Zanotti and Cachanosky ascribe the limited dissemination of Lakatosian versions of praxeology to the strong Rothbardian influence on the praxeological branch of Austrian economics. (2015, p. 132) Whereas Mises’ justifications of praxeology may allow for conflicting interpretations, Rothbard and many of his intellectual followers proudly embrace extreme apriorism. They grant (their respective personal) intuition the role of a truth-criterion. According to Rothbard, a specific form of inner experience allows the social scientist to conceive an apodictic, absolutely certain truth not only about his own mind, but also about the minds of other people^46^: “Man acts.” (Rothbard 1997 (1973), p. 33, pp. 48–49; 1997 (1976), pp. 64–65) Rothbard rejects all alternatives to praxeology as “radically unscientific” (1997 (1960), p. 3) and justifies this verdict extremely aprioristically. Therefore, his justification of praxeology is not compatible with the conventionalism proposed in this paper.

From a Rothbardian stance, this seems to pose a problem for the acceptability of a conventionalist praxeological research program.^47^ However, the arguments provided for a methodology must be distinguished from the ultimate aim of these arguments. According to Rothbard’s own standards, any justification of a methodology is to be appraised by four requirements,^48^ which any fundamental axiom for the social sciences must meet. Ironically enough, these requirements pose more severe difficulties to Rothbard’s own arguments, which heavily depend on intuition, than to a conventionalist justification of praxeology. (Linsbichler 2017, p. 108) Though Rothbard clearly did not endorse conventionalism, perhaps he should have—if he took his own criteria for fundamental axioms seriously.

#### Traces of analyticity and conventionalism in Mises’ defence of praxeology

Section 2.1 outlines the idea and content of praxeology as introduced by Mises and for the most part upheld today. Numerous scholars have identified extreme apriorism as a problem of justifications of praxeology. In Sect. 3.2, we deviate from Mises and argue that a conventionalist justification of praxeology would be congenial to the content of its fundamental axiom. Our proposal is “normative” in the sense that a consistently conventionalist justification has not been provided yet, certainly not by Mises. However, traces of conventionalism can be detected in some of Mises’ own arguments.

Undisputedly, the standard reading of Mises as an extreme synthetic apriorist does have textual support. He professes: “The starting point of praxeology is not a choice of axioms and a decision about methods of procedure (Mises 1998 (1949), p. 39) and “[t]he categories of human thought and action are neither arbitrary products of the human mind nor conventions.” (Mises 1998 (1949), p. 86).^49^ Acknowledging this refusal of conventionalist ideas, the objective of this section is not to show that Mises was a full-fledged conventionalist—he surely was not. Instead, we want to accentuate several hitherto underappreciated remarks and arguments, in which Mises nolens volens approaches a conventionalist defence of an analytic fundamental axiom of praxeology.

Firstly, while Mises uses Kantian terminology and believes in the existence of synthetic judgements a priori (Mises 2012 (1962), p. 5), curiously he never attributes that status to the fundamental axiom or any other sentence of praxeological theory.

Secondly, Mises accepts the existence of alternative, prima facie tenable research programs. (Linsbichler 2017, pp. 83–90) To be sure, Mises also claims that alternatives to the fundamental axiom are unthinkable to the human mind and self-contradictory, hence even “dull people” accept the consequences of the fundamental axiom. (1940, pp. 25–28; 2012 (1962), pp. 4–6, pp. 65–66) In other passages however, these sometimes quite polemic statements are contradicted by Mises himself. He admits that two types of alternatives to praxeology are consistent and cannot be dismissed a priori. (1940, pp. 23–25) Thereby, at least in some moments he grudgingly meets requirement (I) of conventionalism.

While the fundamental axiom states that human individuals act and only human individuals act, prima facie consistent behaviourism denies this by claiming that no objects at all act. Mises as a methodological dualist^50^ argues in a non-metaphysical, but pragmatic way against behaviourism. (1940, pp. 27–28) As a third option, “primitive man” assigns anger to rivers and clouds, and more generally, naive anthropomorphism attributes purposes and ends to a much wider class of objects in nature than the fundamental axiom does (Mises 1940, pp. 24–28).

Mises not only admitted the existence of consistent alternatives to the fundamental axiom. Moreover, his remarks on the history of non-Euclidean geometry (1940, p. 20; 2012(1962), pp. 4–6, pp. 13–21, pp. 63–66) seem to indicate a surmise that alternatives pose a problem to claims of synthetic aprioricity. Admittedly, Mises believed that he ultimately overcame that problem in the case of praxeology and successfully avoided conventionalism.^51^ Still, acknowledging alternatives and the need for further arguments can at least be interpreted as a potential gateway for conventionalism.

Thirdly, Mises ponders that neurophysiologically there might be a continuum between action and non-action. (2003 (1933), pp. 89–90) Still, all praxeological concepts including action either fully apply or do not apply at all. Obviously, the decision, where to draw the clear-cut border involves at least some leeway. This brings Mises closer to a conventionalist position regarding explications of praxeological concepts.

Fourthly, Mises actually propounds purely pragmatic arguments for praxeology:

It is obvious that it is also impossible to demonstrate satisfactorily by ratiocination that the alter ego is a being that aims purposively at ends. [… Praxeology] works, while the idea of dealing with men as if they were stones or mice does not work. It works not only in the search for knowledge and theories but no less in daily practice. (Mises 2005 (1957), p. 165)

At least in these pragmatic arguments, Mises supports requirements of conventionalism by denying conclusiveness (I) and admitting that a proof of the fundamental axiom by pure reason is unattainable (II).

Fifthly, in some instances, Mises is highly critical of intuition as a source of knowledge. Particularly, Mises rejects the claims of certainty and universality attributed to statements arrived at by intuition. To him they have the status of “arbitrary guesses” (2005 (1957), p. 110). Furthermore, Mises raises the problem of intersubjectivity, which he considers an important property of scientific knowledge. Appeals to intuition suffer from “the fact that people can and really do disagree with regard to the interpretation of the inner voice and that no method of peacefully settling such disagreements can be found.” (Mises 2005 (1957), 36) “There is no use arguing with doctrines derived from intuition. […] There is no rational means available for either endorsing or rejecting a doctrine suggested by an inner voice.” (Mises 2005 (1957), p. 110)^52^ These criticisms of intuition, together with the fact that goals and preferences are not directly observable, can be read as further support for requirement (II) of conventionalism.

These traces of conventionalist leanings in Mises’ writings indicate that the standard interpretation of thoroughly extreme apriorism is more problematic than usually recognized. Conversely, the remarks above are by no means sufficient to portray Mises as a full-fledged, self-aware conventionalist. We take the discrepancies^53^ in Mises’ arguments and the ensuing decades of debates on the interpretation of Mises’ defence of praxeology as further corroboration of Scheall’s appraisal that “Mises was thoroughly out of his depths in matters epistemological” (2017b, p. 115). But we do not ask how Mises actually justified praxeology. The more future-oriented focus of this paper is to show that the content of praxeology can in principle be justified without extreme apriorism.

### Conventionalism and its critics: a rejoinder to some misattributions

#### Analytic sentences: trivial, arbitrary, and not universal?

Concluding Sect. 3, three common criticisms of conventionalism should be commented upon briefly: triviality of analytic statements, arbitrariness of conventions, and universality of conventions. After having discussed these objections in turn, we will address a potential ontological worry in Sect. 3.4.2.^54^

“[T]he tautological character of praxeological deduction” (Mises, 1940, p. 19, translated by author) does not imply its triviality or uselessness. Admittedly, analytic statements in a certain sense “cannot add anything to our knowledge.” (Mises 1998 (1949), p. 38). However, just as logic and mathematics, a system of praxeological definitions, deductions, and theorems is potentially highly untrivial and extremely useful for explaining and predicting phenomena in the external world. Analyticity by no means implies triviality.

Conventionalism is sometimes associated with arbitrariness. While in principle linguistic conventions can indeed be constructed at will, the theoretical and practical expediency of linguistic frameworks as a whole and of specific elaborations of definitions within these frameworks has to be tested, critically discussed, and evaluated. Without pragmatically suitable logic, mathematics, and praxeology, it would indeed be much harder or even impossible to build skyscrapers or understand market phenomena. The choice of conventions may and should be guided by experience and interests, but there is no intuitive, observational, or theoretical basis for a straightforward decision between different conventions. Praxeology is designed with a specific purpose, namely epistemological interest in social phenomena. Relative to that (or any other) goal, the choice of a fundamental axiom is not “arbitrary”.

The fundamental axiom and praxeological theorems derived from it are required to be necessary and universal, i.e. praxeological laws “claim validity without respect to place, time, race, nationality, or class of the actor” (Mises 2003 (1933), lxxvii). Although conventions are relative to a language or a research framework, this poses no problem for a conventionalist praxeology because the group of users of a convention has to be distinguished from the group of people who are the universe of discourse, i.e. the subject matter, of the convention. The analytic fundamental axiom does apply universally and is necessary (due to being analytic). Regardless of whether some individuals are ignorant of the fundamental axiom or prefer to use other conventions themselves, the fundamental axiom still claims that they act. Consider for instance anthropomorphists who believe clouds can be angry or behaviourists who (at least in their scientific work) do not think of humans as having intentions and preferences. As praxeologists, we are nonetheless willing and able to apply the universal fundamental axiom to anthropomorphists and behaviourists.^55^ Moreover, we can thymologically account for their (maybe somewhat idiosyncratic) beliefs. Mises makes the same point:

It may be presumed that the Middle Ages would have understood no more of the modern theory of price formation than of Newtonian mechanics or of the modern notions of the functions of the heart. Nevertheless, rain drops fell no differently in the Middle Ages than they do today, and hearts did not beat otherwise than they do now. Though the men of the Middle Ages would not have understood the law of marginal utility, they nevertheless did not and could not act otherwise than as the law of marginal utility describes. […] Even in the Middle Ages no one voluntarily exchanged a horse for a cow unless he valued the cow more highly than the horse. (Mises 2003 (1933), pp. 103–104)

What distinguishes a conventionalist praxeology from its extremely aprioristic counterpart and from a hypothetical “empirical praxeology” is the stance towards the beliefs of anthropomorphists and behaviourists. An extreme apriorist thinks any alternative to her fundamental axiom can be rejected on the basis of intuition, introspection, or some similar faculty. However, the lack of intersubjectivity of these faculties leads to a stalemate when praxeologists and behaviourists have opposing intuitions. An “empirical” praxeologist thinks that observation determines whether clouds can be angry and whether people have preferences—notwithstanding that at least in their customary meaning, emotions and preferences are unobservables. Finally, a conventionalist will accept the prima facie tenability of behaviorists’ and anthopomorphists’ research programs and beliefs, but instead of invoking intuition he will advance pragmatic arguments for the superior expediency of adopting praxeology.

#### Compatibility of scientific realism and conventionalism

Scientific realism and anti-instrumentalism are sometimes considered indispensable tenets of the the Austrian School.^56^ Irrespective of the correctness of this assessment, we will try to demonstrate that a conventionalist justification of praxeology is perfectly compatible with these, but also with other ontological and epistemological positions.

Given the plethora of different definitions and connotations of “realism”, “anti-realism”, and “instrumentalism” we start by slightly specifying these concepts. At first, note that in our context the debate between realism and ant-realism is not concerned with the existence or non-existence of observable objects, such as desks, swans, and humans. The question is rather, what the theoretical terms in our theories refer to and whether they refer to anything at all. If terms like “electron”, “field”, or “preference” refer to something, these objects are not directly observable. A typical anti-realist denies that theoretical terms refer to anything; consequently, sentences containing theoretical terms have no literal meaning and hence no truth-values. Therefore, anti-realism is committed to an instrumentalist view regarding theoretical terms.^57^ “According to the instrumentalist view, a theory is not really to be believed to give us a truthful picture of what the world is like, it is rather to be used as a useful tool for whatever purposes there may be.” (Mäki 2001, p. 10)^58^ Conversely, instrumentalism does not entail anti-realism. For an instrumentalist, the sentences of her favourite theory could very well have literal meaning and truth-values. However, she possibly upholds a theory without caring that it is known to be false. Conflation of these two variants of instrumentalism sometimes triggers misunderstandings: To put it in a nutshell, anti-realist instrumentalists deny that certain sentences have literal meaning and truth-values, whereas “indifferent” instrumentalists do not care whether theories are true or false.

We are not emphatically interested in establishing that praxeological theory could be interpreted in an anti-realist or instrumentalist way. Our goal in this section is rather to assure those scholars who deem realism and anti-instrumentalism generic and indispensable tenets of the Austrian School that these positions are compatible with conventionalism as characterized in Sects. 3.1 and 3.2.^59^

To begin with, note how any conventionalist endeavour is profoundly concerned with the truth-values of sentences. Thus, conventionalism is a (moderately) aprioristic and not an instrumentalist (pseudo-sentence) position.^60^ Otherwise the effort to keep some truth-values fixed would be pointless. Indeed, the deep sentiment of Poincaré’s most famous conventionalism in physics ultimately sounds realist, not instrumentalist, as well:

When a law has received a sufficient confirmation from experiment, we may adopt two attitudes: Either we may leave this law in the fray; it will then remain subject to incessant revision […]. Or else we may elevate it into a principle by adopting conventions. (Poincaré 1982 (1905, 1913), p. 335)

Turning to conventionalist praxeology, recall that the modern debate between realism and anti-realism is restricted to the unobservable realm. Any realist concerns with the fundamental axiom presuppose that its subject matter are *theoretical* entities. For the realist, the fundamental axiom describes these “really” existing theoretical entities or their structure. Since theoretical entities are unobservable, direct confirmation or falsification by observation is barred beforehand. If the realist refrains from taking recourse to intuition in her justification of the fundamental axiom, requirement (II) of conventionalism can be fulfilled easily: *The conventions are not justified by observation or intuition, but by pragmatic arguments for the superior expediency of the resulting theory or research program*.^61^

Lastly, a similar argument can be given for requirement (I) of conventionalism: *The conventions could in principle have been chosen differently, i.e. alternative theories or research programs are possible.* Again, a realist presumes that the fundamental axiom refers to unobservables. In this case, a modest fallibilism suffices to accept alternatives to a specific fundamental axiom. Accordingly, an eminent proponent of realism for theories of Austrian economics concludes: “[T]he existence of such rival systems can be seen to be a perfectly natural and acceptable consequence of the just-mentioned difficulties we will often face in coming to know even the intelligible traits of reality” (Smith 1996, p. 191). A reasonable realist carefully distinguishes between the belief that certain unobservable entities “really” exist and the pretension that he possesses the certified, unique, true theory about these unobservables.

We affirm simply that there are synthetic intrinsically plausible true propositions, and that science strives to accumulate ever more of these; we do not however affirm that we know (or much less that we have certain knowledge about) which of the available candidates for such propositions are true among those which at any given time play a role in the really existing sciences. The given intelligible structural traits of reality can be overlooked or misinterpreted. The recognition that there are a priori structural traits in the world yields, to repeat, no easy sort of indubitable evidence in relation to the corresponding propositions. (Smith 1996, p. 191)

The principal idea of a conventionalist defence of praxeology is to accept that observation is of no avail for the fundamental axiom and to reject intuition as a reliable criterion of truth. As a consequence, justifications of the fundamental axiom must ultimately be pragmatic. Anti-realist as well as realist praxeologists search for a fundamental axiom with great pragmatic success. While anti-realists or scholars who regard many aspects of the debates between realists and anti-realists as pseudo-problems are satisfied with this, realists aim at an additional conclusion. Realists argue that pragmatic success of one version of the fundamental axiom suggests that this version depicts “really” existing structures of unobservables—“the real structure of human action”.^62^ Such a typical inference to the best explanation might be portrayed as well justified reasoning by realists and as a speculative guess by anti-realists.^63^ With regard to this disagreement, conventionalism is silent. Adopting conventionalist praxeology is perfectly consistent for realists and anti-instrumentalists in the Austrian School.

## Conclusion: The prospects of conventionalist praxeology

### Methodologies and their justifications

Among praxeologists there is a fair amount of consensus as to the content of the fundamental axiom and the implementation of praxeological methodology as introduced by Mises and portrayed in Sect. 2. Open problems and “puzzles” are solved by means of what we might call “normal economic science”. In contrast, seemingly irreconcilable disagreements separate various different justifications of praxeology. For instance, Rothbard (1998), Hoppe (1983, 1995), Long (2013), and others provide their own justifications of the fundamental axiom. The philosophical backgrounds and arguments of these authors sometimes considerably deviate from the standard reading of Mises. On top of that, Mises’ own writings contain mutually exclusive deliberations about the epistemological status of the fundamental axiom.

Over and above the discordance within the Austrian School, all the prominent justifications of praxeology are almost unanimously considered epitomes of extreme apriorism. While our paper does not argue why extreme apriorism is inferior to conventionalism but presupposes this as a standard of contemporary philosophy of science, the well-substantiated demise of extreme apriorism’s respectability prima facie poses a serious problem for praxeology.

Presumably with praxeology in mind, the prime methodologist of the Mises circle already emphasized that oftentimes “good methods are ‘justified philosophically’ with bad arguments” (Kaufmann 2014 (1936), p. 106). Similarly, Hayek expressed his discomfort with some of Mises’ less convincing arguments, but at the same time agreed with most of the conclusions. (Hayek 1978b, 2009 (1978)) In Kaufmann’s and Hayek’s spirit, this paper seeks improved justifications of praxeology.

In Sect. 3, we argue that a relatively uncontroversial version of conventionalism is sufficient to obtain analytic truth for the fundamental axiom. Withal, the content and implementation of praxeology remains untouched by these epistemological considerations. Logical deductions of new praxeological theorems can be attempted just like before and explanations and predictions of social phenomena can be provided just like before, i.e. using praxeology, thymology, and possibly logic, mathematics, and the natural sciences.^64^

In comparison to the extremely aprioristic justifications of praxeology hitherto dominant, our conventionalist alternative is even less aprioristic with respect to the *content* dimension (i). An already very narrow scope of the fundamental axiom is further reduced to vacuity. More importantly, the *kind of justification* (ii) is not extremely aprioristic anymore. Neither Hutchison (1998) nor Scheall (2017a) classify truth by convention as extremely aprioristic. Indeed, one of the main achievements of philosophy of science of the last century was the elimination of intuition and synthetic a priori judgements as a necessary basis for the justification of logic, mathematics, and empirical sciences. With some delay, a similar possibility has now been made explicit for praxeology. Finally, the *certainty* (iii) of analytic praxeological theorems is of course higher than the certainty of almost all empirical hypotheses in the natural sciences. However, the adequate comparison is rather between praxeology and definitional and mathematical auxiliary constructions in other disciplines. In that case, the respective certainties exculpate praxeology of extreme apriorism regarding (iii).

From the perspective of mainstream philosophy of science and mainstream economics, we expect the moderation in apriorism to increases the acceptability of praxeology immensely.^65^ Following a clearance of extreme apriorism, a main novelty accompanying the proposed shift from synthetic to analytic praxeology would be a call for communication and comparison with other schools of thought. We hope our conventionalist proposal contributes to an encouragement of more constructive discussions within the Austrian School as well as between the Austrian School and other research programs.

A closely related benefit of the conventionalist version of praxeology is a raised compatibility with the use of formal methods. Congruously with his rudiments of conventionalism, Mises only rejects certain inadmissible applications of mathematical methods, whereas Rothbard, who has no conventionalist leanings whatsoever but champions an essentialist theory of meaning, wholeheartedly rejects the use of formal methods in economics.^66^

Furthermore, several authors have conjectured that the adoptions of epistemological or methodological positions and of political worldviews are not independent of each other (see e.g. Boettke 1995; Popper 1975, 1980; Talmon 1985a, b). Keeping that in mind, a justification of praxeology should ideally be in line with the political liberalism defended by many Austrian economists. Accordingly, Scheall (2015c) champions “methodological liberalism” at the foundation of the Austrian School. When confronting the complex problem of evaluating research programs, his “methodological liberalism” advocates a form of humble pluralism instead of irreversibly discarding all but one research program on allegedly neutral and universally valid grounds, as some extremely aprioristic praxeologists espouse.

Since praxeological methodology rules out observation as a critical standard anyhow, this paper proposes to adopt a form of conventionalism as an auspicious strategy to avoid dogmatic tendencies. The epistemological openness and modesty of conventionalism ideally match the plea for political openness and modesty dear to many Austrian scholars.

### A double-edged outlook

Praxeology, the methodology of the Misesean branch of the Austrian School, is considered a particularly extreme example of apriorism in economics. In this paper, we argued that this assessment only applies to the justifications of praxeology hitherto prevalent. In comparison, a possible and plausible conventionalist justification takes the fundamental axiom of praxeology to be analytic instead of synthetic, thereby yielding a moderately aprioristic version of Austrian economics.

Given a conventionalist justification of praxeology, the economic thought of the Austrian School cannot be straightforwardly rejected on epistemological grounds anymore. Austrian positions in the calculation debates, regarding entrepreneurial discovery and thoroughgoing subjectivism, or in capital theory and by implication in explanations of the business cycle could gain respectability. Non-Austrian economists would perhaps continue enhancing their attention to the role of information, prizes, institutions, and time in the market process.

The flipside of this point is, however, that in turn Austrian economists are called upon to extend the humbleness they display when it comes to quantitative predictability and arbitrary tractability of social phenomena.^67^ Particularly, this paper prompts praxeologists to accept the prima facie tenability of competing research programs aand their results. Instead of invoking pure intuition as an allegedly infallible truth-criterion to declare all other research programs fallacious, the relative scientific expediency of slight variations of praxeology and of praxeology against entirely different approaches should then be critically discussed and fastidiously evaluated. Even more generally speaking, we believe increased explicitness about the methodological status and about the exact content of antithetical first principles can contribute to turning a partisan stalemate into a more nuanced, fruitful discussion. In science and beyond.

## Footnotes

1 Orthodox models using unrealistic assumptions may of course self-consciously serve other purposes than providing realistic descriptions.

2 Linsbichler (2017, pp. 35–36) provides a systematic overview of epistemological positions rejected by Mises and his reasons for doing so.

3 Note that Mises’ rejection of experience as a critical standard for praxeology is on its own insufficient for substantiating his alleged dogmatism and infallibilism. As for the latter, he knows that praxeology is subject to error just like any human endeavour (Mises 1998 (1949), p. 68); but the critical standard is similar to mathematics: “The only way to refuse a conclusion of economics is to demonstrate that it contains a logical fallacy” (Mises 1990 (1942), p. 9).

4 As a special case of experience, Boianovsky (2018) accurately portrays Mises as attributing little to no value to travelling, theorywise. It should be kept in mind, however, that Mises uses the terms “theory” and “economics” in an idiosyncratic and narrow way. They refer to the deductive inferences from the a priori true starting point only. Social scientific knowledge in a broader sense can of course be improved by travelling and other empirical research, even according to Mises.

5 The explication of the fundamental axiom is, however, an ongoing task. Analogous to axiomatizations in other disciplines, we would expect progress from the utilization of modern formal logic.

6 As an instructive example consider knee jerk: When tested by a physician, it will usually be considered an action; in comparison, when knee jerk is a reflex to stumbling it is usually inadequate to consider it an action, because the almost stumbling individual had no goal or intention “to let the knee jerk happen”, let alone “to activate a knee jerk”. Hence, the identification of action with conscious behaviour may have some heuristic value but is subject to severe restrictions.

7 Suffice to say that Quine (1951) did not mark the end of conventionalism or of the distinction between analytic and synthetic. Creath (1987, 1991), Alspector-Kelly (2001), Russell (2008), Psillos (2011), Friedman (2012), and many others substantiate that the debate is still unresolved. Only 27.1% of the philosophers in a survey conducted by Bourget and Chalmers follow Quine in rejecting the analytic-synthetic distinction (2014, p. 476). Sidelle (1989) provides a post-Quinean defence of necessary truths by convention.

8 Among the distinguishing marks of extreme apriorism are claims that observation of the external world (outer experience) plays little or no role in the justification of scientific knowledge. Instead, inner experience (intuition or introspection) or pure reason are granted a privileged role.

9 See (Hutchison 1998) for a history of ultra-deductivism. His major point of criticism against apriorist positions is their use of unrealistic claims about the knowledge of the acting individuals and the ensuing problems to discuss disequilibria. But the fundamental axiom states neither perfect information nor rational expectations. On the contrary, the central role of imperfect and dispersed information in the approach of the Austrian School has been manifest ever since (Hayek 1948 (1937)) at the latest, but probably even earlier, as Kirzner (2001, pp. 86–87) convincingly argues.

10 For the auxiliary axioms see (Rothbard 1997 (1957), pp. 102–103; White 2003 (1977), p. 16; Linsbichler 2017, pp. 45–46).

11 Strictly speaking, sentences the deduction of which requires not only the fundamental axiom but also at least one auxiliary axiom, can be called praxeological theorems and a priori using a trick. Analogously to the classification of mathematics as a part of logic in the tradition following Russell, most praxeological theorems would have the form “If auxiliary axioms A_1_, A_2_, … obtain, then p.”.

12 Fallenstein (2017) provocatively labelled the tension between libertarian content and dogmatic conduct “libertarian Stalinism”.

13 See Linsbichler (2017, p. 48, pp. 52–56, pp. 97–98) for a more detailed account and examples of the interplay between praxeology and thymology.

14 The narrowness of the aprioristic fundamental axiom has indeed raised criticism. Among others, Zilian (1990), Schröder (2010), and Nelson (1992) doubt whether such a restricted basis is sufficient for the far-reaching asserted conclusions. While these skeptical authors may have a point regarding gaps in the deductive chain of praxeological theorizing, the role of thymology in Mises’ conception of Austrian economics should not be underestimated. „It is of course true enough that praxeology will avail us little unless we know how to apply it, and that there is no apodictically certain method of applying it. That is not an objection to Mises’ doctrine; it is Mises’ doctrine.” (Long 2013, p. 58).

15 For discussions of the demonstrated preference approach see (Rothbard 1997 (1956); Gordon 1993; Hudik 2012; Pham 2017). However, these excellent expositions do not focus on the necessary thymological component. That is why they suggest an understatement of the subjectivity and related problems of every application of Verstehen.

16 For the case of Mises, see (Scheall 2017a); for aprioristic positions in the history of economic methodology see (Hutchison 1998). Given Robbins’s (1932) paramount and lasting impact on the discipline and considering that his apriorism unequivocally relies on intuition, the orthodoxy’s comparatively harsh opposition to Mises’ apriorism is somewhat unexpected. Latsis (1976, pp. 4–5) provides additional indication of imbalance in the mainstream’s assessment. He argues that most economists and methodologists of economics have in fact tacitly adopted Mises’ praxeological methodology.

17 Having said that, the gist of Tokumaru’s proposal does point at the conventionalist justification of praxeology taken in Sect. 3.2. Like in the case of a rationality principle of situational analysis discussed by Tokumaru, conventionalist praxeologists follow a methodological rule to the effect that the fundamental axiom is held true irrespective of any observational data. (Linsbichler 2017, pp. 75–78).

18 Linsbichler (2017, pp. 79–80) contends that context in which Mises discusses notions of genetic or psychological is not the aprioricity of the fundamental axiom, but either the fallibilism of every human endeavour including science (2005 (1957), p. 49) or the attempt (contra Wieser, Mayer, and others) to explicitly de-psychologize economics (Mises 2003 (1933), pp. 144–145; 1990 (1944), p. 21; 1998 (1949), pp. 84–87; 2005 (1957), p. 202; 2012 (1962), p. 4, p. 8, pp. 13–18; 1940, p 20).

19 Elements of the idea of justifying praxeology as a genetic or psychological a priori can be found in the otherwise highly commendable (Leeson and Boettke 2006). They argue that the praxeological concepts developed in the human mind and will develop further. This evolutionary process renders the fundamental axiom true and apodictically certain, say Leeson and Boettke (2006, pp. 256–257). However, their instrumentalist notion of truth rests on a pragmatist background which is alien to Mises. See e.g. Mises (2005 (1957), p. 182).

20 Although evolutionary epistemology cannot justify a priori truth of the fundamental axiom, its insights into the evolutionary contingency of human beliefs, values, and expectations have played an eminent role in the Austrian School ever since Hayek (1999 (1952)) at the latest.

21 Of course, there are numerous different theories of action even outside the Austrian School that bear the name “praxeology” or “praxiology”. Other theories of action resemble Mises’ approach by content, but not by name. Accordingly, there is considerable disagreement in the secondary literature, who represents praxeology and who does not. However, Mises and Rothbard are unanimously regarded as main proponents of the Austrian praxeological research program.

22 On a different, though related point, Caldwell controverted Hutchison by highlighting Popperian elements in Hayek’s position. See (Caldwell 2009) for a retrospective of the debate. Milford (2001 (1994)) by and large concurs with Caldwell; Schröder (2010, p. 216) points to one of several opposing passages in Hayek’s writings.

23 Notwithstanding Hayek’s intentions and the received view, Mises applauded Hayek’s contribution as consistent with his praxeology. (Zanotti and Cachanosky 2015, p. 125).

24 Empirically minded commentators might recognize this limited reach of a priori reasoning as a merit of Hayek’s position. In addition, Hayek’s appraisal of the empirical reach can be reconstructed as equally modest: “[W]hatever knowledge we possess with respect to economic phenomena comes to us via experience and that experience doesn’t furnish a great deal of knowledge with respect to economic phenomena” (Scheall 2015a, p. 101).

25 See e.g. Oakley (2000, 2002), Kurrild-Klitgaard (2001, 2003), and Helling (1988).

26 See Linsbichler (2019).

27 Confer footnote 41.

28 Confer footnote 36.

29 Prominent resources of our characterization include Wright (1957, pp. 40–53), Sidelle (1989), Popper (2010 (1930–1933), pp. 221–245), Poincaré (1982 (1905, 1913)), Frank (1957), and Carnap (see Creath 1987, 1991; Psillos 2011; Friedman 2012).

30 See also Blaug (1985, pp. 702–705).

31 Technically, one option to achieve this would be by using Hilbert’s ε-operator. There is a close relationship between the ε-approach to reconstructing theories and a structuralist interpretation of theoretical terms. This should gratify the many realists in the Austrian School who, like Smith (1996), think that "[p]raxeology strips action down to its barest formal structure" (Martin 2015, p. 18).

32 Pragmatic virtues include but are not restricted to predictive power, forms of simplicity, explanatory power, resemblance of nomenclature to everyday language.

33 Of course, the concept of observability itself entails various open problems in philosophy of science. We leave it at adverting to the benefits of a conventionalist approach to observability itself. See (Dicken 2013).

34 See also (Mises 2003 (1933), p. 14; Linsbichler 2017, p. 108; Rothbard 1997 (1976), p. 69) for perceived parallels between mathematics and praxeology.

35 For the sake of argument, we assume this claim of monetary theory is actually analytic, i.e. deducible from the fundamental axiom and definitions of the terms involved. Examinations of the alleged chain of deductions for implicit and unnoticed non-analytic auxiliary assumptions are certainly judicious.

36 To many Austrian economists who challenge praxeology it comes even more naturally not to a priori rule out any potentially observable states of affairs (III). What we praise as a predisposition for uncontroversial conventionalism prompts harsh criticism by a self-consciously extremely aprioristic praxeologist: “Hayek […] Kirzner […] and Lachmann [… view …] economics as some sort of science of human knowledge. Accordingly, Hayek’s categories and theories refer to purely subjective phenomena and are invariably elusive or even illusory. He is not concerned about acting with things but about knowledge and ignorance, the division, dispersion, and diffusion of knowledge, alertness, discovery, learning, and the coordination and divergence of plans and expectations. The external (physical) world and real (material) events have almost completely disappeared from his view. Hayek’s categories refer to mental states of affairs and relationships, completely detached from and compatible with any real physical state of affairs and events.” (Hoppe 2006, p. 260).

37 One hypothetical caveat is Mises’ insinuation of a state of far advanced neurophysiology, in which a decision between praxeology and behaviourism could be approached. Mises suspects the fundamental axiom could be construed as having minimal empirical content provided that the content of human thoughts could be read directly from the observable physical and chemical processes in the human brain. (1978 (1940), p. 85).

38 See (Carnap 1968 (1934), p. 246) for a clarification of the often-overlooked possibility to revise analytic concepts (“L-rules”) or to switch to an entirely new linguistic framework.

39 The implicit ontological dimension of Mises’ praxeology is e.g. discerned by Oakley (1997).

40 If the ability to act is linked with so-called natural rights, as in Rothbard’s ethic (1998), the question who acts becomes even more relevant—and extreme apriorism politically dubious. It is hard to see how cases like unborn children, infants, aliens, and animals can be decided unanimously by intuition or pure reason as non-conventionalist natural law doctrine suggests.

41 Schütz, Hayek, Shackle, Lachmann, Lavoie, and others have criticized basic assumptions of Mises’ economics and ultimately arrived at different conclusions regarding, for instance, the equilibrating tendencies of economic systems. See e.g. Klein (2012), Langlois (1982), Selgin (1990), and Rizzo, who includes "There is a tendency toward coordination of individual activities." in the hard core of Austrian economics. He claims that coordination can be derived from the fundamental axiom. (1982, pp. 57–58). It is not obvious whether these differences can be traced back to deviations in the aprioristic details of explicating the concept of action or to other, non-aprioristic differences. Note that for sake of this comparison, we disregarded the alternative concept of a priori which some of these authors defend. It is ideal–typical and hence lacking the necessity of a fundamental axiom.

42 Some proponents of an analytic fundamental axiom do not take the step to conventionalism. The so-called *Hamburger Deutung* (Hamburg interpretation) interprets praxeology as analytic, i.e. true in virtue of meaning, as well. The conceptual analysis Puster (2014) and Oliva Cordoba have in mind is, however, hardly concerned with “the obvious question, how a purely logical deduction from aprioristic principles can tell us anything about reality” (Mises 1990 (1942), p. 9). Instead of examining the relation of the fundamental axiom to the external world, Oliva Cordoba (2017) commendably uses modern formal logic to analyse the concept of action. While we fully agree with the logical and explicatory aspect of this project, it seems the *Hamburger Deutung* denies the dependence of intersubjective concepts on language. As a consequence, its ultimate aim is not the explication of expedient terminological conventions but the discovery and establishment of *the* allegedly unique concept of action. This idea is most probably more akin to Wieser’s (1884, pp. 1–41) quest for the Sprachgeist than to praxeology.

43 See Lakatos (1978), Backhouse (1998).

44 Similarly, Zanotti and Cachanosky (2017, p. 118) include the prima facie synthetic disutility of labour in the hard core, without however claiming derivability from the fundamental axiom of purposeful action.

45 Langlois (1982) continues this line of inquiry by providing insightful contrasts between variants of Austrian economics with neoclassical economics.

46 Rothbard maintains that intuition reveals that all humans and only humans act. Even if we grant that the intuition of a person x can reveal to x that x acts, it is highly questionable at best how this claim should be (intersubjectively) extended to all other persons as Rothbard himself requires. While Rothbard and Mises share praxeological and thymological methodology, their philosophical backgrounds and their justifications of the fundamental axiom differ. Confer Mises’ (2003 (1933), p. 42; 1940, pp. 17–19; 2005 (1957), p. 110) reservations regarding the role of inner experience.

47 For the most part, this paper takes the superiority of conventionalism over extreme apriorism for granted, based on the standards of contemporary philosophy of science. However, Rothbardian praxeologists might hold different evaluative standards. These praxeologists might now and then embrace some conventionalist arguments strategically, but ultimately champion extreme apriorism in spite of its (well-reasoned) demise in academic philosophy. Even so, we maintain that an import of “external” evaluative standards might even be expendable in this case. Rothbard’s own “internal” standards suffice to support a conventionalist defence of praxeology by means of an “undogmatic methodological critique” (Caldwell 1984, p. 129).

48 Rothbard’s four principal requirements for being eligible as a fundamental axiom for the social sciences as rationally reconstructed from (1997 (1976), pp. 63–65; 1997b (1957), pp. 105–106) arrogate: The fundamental axiom needs to be “empirically meaningful” (in an idiosyncratically weak sense), “a priori to complex historical events”, “not conceivably falsifiable”, and “absolutely true” (where the intended difference between truth and absolute truth remains cryptic).

49 Confer Section 2.3 for further elements of genetic or psychological apriori in Mises’ writings.

50 For the methodological character of Mises’ dualism see (Linsbichler 2017, pp. 13–16).

51 Linsbichler (2017, pp. 83–88) argues that all of Mises’ respective arguments conflict with positions he advocates elsewhere.

52 See also (Mises 2003 (1933), pp. 46–48).

53 For an account of discrepancies in Mises defence of praxeology see Linsbichler (2017, pp. 73–91).

54 We thank an anonymous reviewer for raising the objections re universality and ontology. See also footnote 58.

55 Block (1980, p. 431) makes a related point about the proposition that all actions are rational. Praxeologists methodologically decide to adopt this as an analytic convention because they want their “research manifesto” to apply universally to all actions. If there were irrational actions, they could not be explained praxeologically.

56 See e.g. Caldwell (1992), Hoppe (1995, pp. 19–22, pp. 69–70), Mäki (1992), and the references given by Hülsmann (1999, footnote 1). Although Hülsmann’s claim that the Austrian School “has consistently adhered to the postulate of economic realism” (1999, p. 3) might be slightly overstated (see e.g. Caplan 1999, 2001), at least for many praxeologists realism and anti-instrumentalism are serious concerns. We are grateful to two anonymous reviewers for sharing their - mutually exclusive - in-depth appraisals of realism in the Austrian School.

57 As an example, Milford and Cerman (2011) rationally reconstruct Schumpeter’s epistemological position as a pseudo-sentence position and point to problems of the resulting instrumentalism. Pseudo-sentences only appear to be sentences but are actually sentential functions. Hence, they have no truth-value.

58 One of several hazards of instrumentalism which apprehending Austrian economists are concerned about is the following: The “purposes” relative to which instrumentalists evaluate and apply their scientific theories need not be epistemic purposes. Instead of weighing different epistemic virtues, instrumentalism grants political bias an easy gateway, notably in a discipline like economics.

59 One anonymous reviewer has a different definition of conventionalism in mind: “Conventionalism is the idea that there are fundamental principles grounded on agreements in society (or in the scientific community), rather than on external reality.” This position seems to resemble a form of social constructivism and may indeed be inconsonant with realism and anti-instrumentalism. But conventionalism with an emphasis on the second immunizing strategy as portrayed in Sect. 3.1 is fully compatible with realism. For a meticulous and compelling depiction of Carnap’s conventionalism as neutral between realism and instrumentalism see (Friedman 2012).

60 See Popper (2010 (1930–1933)), Milford (1992), Linsbichler (2017, pp. 57–72).

61 Requirement (III) for the uncontroversial variant of conventionalism is met trivially: *Conventions are restricted to sentences that do not exclude any potentially observable states of affairs.*

62 Reversely, any attempt to justify a specific fundamental axiom by merely claiming the existence of unobservable structures is a non-sequitur. A realist stance regarding the “structure of action” is by itself of no avail for determining the actual shape of that structure. More specifically, without direct access to unobservable structures, nothing concrete about the fundamental axiom can be deduced.

63 Using a heuristic gauge, we could compare inferences to the best explanation to other pragmatic arguments. If unobservable objects exist, professing their structure by inference to the best explanation seems less pragmatic than claiming that structures in the real world must be simple, which in turn seems much less pragmatic than purporting that structures in the world accommodate my personal wishful thinking. Inferences to the best explanation are presumably among the least pragmatic arguments, but they are still pragmatic as a comparison with justification by direct observation shows.

64 In fact, praxeological laws being analytic ideally fit the idea of avoiding their falsification by putting all empirical content into the boundary conditions. See Linsbichler (2017, pp. 52–55).

65 Conventionalism might not be the only strategy to conciliate praxeology with modern philosophy of science. The paper does not claim that conventionalism is ultimately preferable to, for instance, modern very modestly aprioristic Neo-Kantian approaches. For instance, Friedman’s (2001) historicized version of a relativized priori may be eligible, but whether it could actually be applied to praxeology in a congruent manner remains to be shown.

66 See also (Caldwell 1984; Egger 1978; Kaufmann 1930; Menger 1972; Hudik 2015; Linsbichler 2017, pp. 99–105, 110–112).

67 See also Dekker (2016, pp. 109–130).

## References

[CR1] Alspector-Kelly M (2001). On Quine on Carnap on ontology. Philosophical Studies.

[CR2] Backhouse R (1998). Explorations in economic methodology: From Lakatos to empirical philosophy of science.

[CR3] Blaug M (1985). Economic theory in retrospect.

[CR4] Blaug M (2006). The methodology of economics: Or how economists explain.

[CR5] Block W (1980). On Robert Nozick’s ‘On Austrian Methodology’. Inquiry.

[CR6] Boettke P (1995). Why are there no Austrian socialists? Ideology, science and the Austrian School. Journal of the History of Economic Thought.

[CR7] Boianovsky, M. (2018). Economists and their travels, or the time when JFK sent Douglass North on a mission to Brazil. *Journal of the History of Economic Thought*, 40, forthcoming.

[CR8] Bourget D, Chalmers D (2014). What do philosophers believe?. Philosophical Studies.

[CR9] Cachanosky, N. (2014). *Rejoinder to David Gordon*. https://puntodevistaeconomico.wordpress.com/2014/04/03/rejoinder-to-david-gordon/.

[CR10] Caldwell B (1984). Praxeology and its critics: An appraisal. History of Political Economy.

[CR11] Caldwell B, Caldwell B, Böhm S (1992). Commentary. Austrian economics: Tensions and new directions.

[CR12] Caldwell B (2009). A skirmish in the Popper Wars: Hutchison versus Caldwell on Hayek, Popper, Mises, and methodology. Journal of Economic Methodology.

[CR13] Caplan B (1999). The Austrian search for realistic foundations. Southern Economic Journal.

[CR14] Caplan B (2001). Probability, common sense, and realism: A reply to Hülsmann and Block. The Quarterly Journal of Austrian Economics.

[CR15] Carnap, R. (1968 (1934)). *Logische Syntax der Sprache* (Zweite, unveränderte Ausg.). Wien: Springer.

[CR16] Creath R (1987). The initial reception of Carnap’s doctrine of analyticity. Nous.

[CR17] Creath R (1991). Every dogma has its day. Erkenntnis.

[CR18] Dekker E (2016). The Viennese students of civilization: The meaning and context of Austrian economics reconsidered.

[CR19] Di Iorio F (2008). Apriorism and fallibilism: Mises and Popper on the explanation of action and social phenomena. Nuova Civilta della Macchine.

[CR20] Dicken P (2013). Tolerance and voluntarism. Philosophical Papers.

[CR21] Egger JB, Spadaro LM (1978). The Austrian method. New directions in Austrian economics.

[CR22] Fallenstein, D. (2017). *Libertarian Stalinism is a thing*. https://ftn.media/libertarian-stalinism-thing.

[CR23] Frank P (1957). Philosophy of science. The link between science and philosophy.

[CR24] Friedman M (2001). Dynamics of reason.

[CR25] Friedman M, Frappier M, Brown D, DiSalle R (2012). Carnap’s philosophical neutrality between realism and instrumentalism. Analysis and interpretation in the exact sciences.

[CR26] Gordon D (1993). Toward a deconstruction of utility and welfare economics. The Review of Austrian Economics.

[CR27] Gordon, D. (2014). *Mises and the diminished a priori*. http://mises.org/daily/6711/Mises-and-the-Diminished-apriori.

[CR28] Hausman D (2011). Preferences, value, choice, and welfare.

[CR29] Hayek, F. A. (1948 (1937)). Economics and knowledge. In *Individualism and economic order* (pp. 33–56). Chicago: The University of Chicago Press.

[CR30] Hayek, F. A. (1978a). *Interview with Axel Leijonhufvud*. https://ia801407.us.archive.org/18/items/nobelprizewinnin00haye/nobelprizewinnin00haye.pdf .

[CR31] Hayek FA (1978). Coping with ignorance. Imprimis.

[CR32] Hayek, F. A. (1999 (1952)). *The sensory order: An inquiry into the foundations of theoretical psychology*. Chicago, IL: University of Chicago Press.

[CR33] Hayek, F. A. (2009 (1978)). Introduction. In: L. Mises (Ed.), *Memoirs* (pp. xiii–xx). Auburn: Mises Institute.

[CR34] Helling I, List E, Srubar I (1988). Alfred Schütz, Felix Kaufmann, and the economists of the Mises circle: Personal and methodological continuities. Alfred Schütz, neue Beiträge zur Rezeption seines Werkes.

[CR35] Hoover, K. (2017). First principles, fallibilism, and economics (January 1, 2017). *The Center for the History of Political Economy Working Paper Series*, 2017-01. Available at SSRN: https://ssrn.com/abstract=2901539.

[CR36] Hoppe, H. (1983). *Kritik der kausalwissenschaftlichen Sozialforschung: Untersuchungen zur Grundlegung von Soziologie und Ökonomie*. Studien zur Sozialwissenschaft 55. Opladen: Westdt. Verl.; VS Verlag für Sozialwissenschaften.

[CR37] Hoppe H (1995). Economic science and the Austrian method.

[CR38] Hoppe H (2006). The ethics and economics of private property.

[CR39] Hudik M (2012). Transitivity: A comment on Block and Barnett. The Quarterly Journal of Austrian Economics.

[CR40] Hudik M, Bylund PL, Howden D, Salerno JT (2015). ‘Mises and Hayek Mathematized’: Toward mathematical Austrian economics. The next generation of Austrian economics: Essays in honor of Joseph T. Salerno.

[CR41] Hülsmann JG (1999). Economic science and neoclassicism. The Quarterly Journal of Austrian Economics.

[CR42] Hutchison TW (1981). The politics and philosophy of economics: Marxians, Keynesians, and Austrians.

[CR43] Hutchison TW (1998). Ultra-deductivism from Nassau Senior to Lionel Robbins and Daniel Hausman. Journal of Economic Methodology.

[CR44] Kaufmann F (1930). Was kann die mathematische Methode in der Nationalökonomie leisten?. Zeitschrift für Nationalökonomie.

[CR45] Kaufmann F, Cohen R, Helling I (1936). Theory and method in the social sciences. Felix Kaufmann’s theory and method in the social sciences.

[CR46] Kirzner I (2001). Ludwig Von Mises: The man and his economics. Library of Modern Thinkers.

[CR47] Klein, P. G. (2009). *A note of Giffen goods* (revised 5 Nov 2009). https://cpb-us-w2.wpmucdn.com/sites.baylor.edu/dist/a/122/files/2016/06/giffen-t82d17.pdf.

[CR48] Klein DB (2012). Knowledge and coordination. A liberal interpretation.

[CR49] Kurrild-Klitgaard P (2001). On rationality, ideal types and economics: Alfred Schütz and the Austrian School. The Review of Austrian Economics.

[CR50] Kurrild-Klitgaard P (2003). The Viennese connection: Alfred Schutz and the Austrian School. The Quarterly Journal of Austrian Economics.

[CR51] Lachmann, L. (1956). *Letter to Ludwig von Mises*, 17.9.1956, Archive of the Mises Institute, Auburn, Alabama.

[CR52] Lakatos I (1978). The methodology of scientific research programmes.

[CR53] Langlois R, Kirzner I (1982). Austrian economics as affirmative science: Comment on Rizzo. Method, process, and Austrian economics: Essays in honor of Ludwig von Mises.

[CR54] Latsis S, Latsis S (1976). A research programme in economics. Method and appraisal in economics.

[CR55] Leeson P, Boettke P (2006). Was Mises right?. Review of Social Economy.

[CR56] Linsbichler A (2017). Was Ludwig von Mises a conventionalist?—A new analysis of the epistemology of the Austrian School of economics.

[CR57] Linsbichler, A. (2019). Felix Kaufmann—‘A Reasonable Positivist’?. In F. Stadler (Ed.), *Ernst Mach*—*Life, work, influence; Vienna Circle Institute Yearbook 22* (forthcoming).

[CR58] Long R (2013). Wittgenstein, Austrian economics, and the logic of action—Praxeological Investigations.

[CR59] Mäki U (1992). The market as an isolated causal process: A metaphysical ground for realism. Caldwell and Böhm.

[CR60] Mäki U, Mäki U (2001). Economic ontology: What? Why? How?. The economic world view. Studies in the ontology of economics.

[CR61] Martin A, Boettke P, Coyne CJ (2015). Austrian methodology: A review and synthesis. The Oxford handbook of Austrian economics.

[CR62] Menger K (1972). Österreichischer Marginalismus und mathematische Ökonomie. Zeitschrift fiir National6konomie.

[CR63] Milford K (1992). ‘Poppers Lösungsvorschlag des Abgrenzungsproblems und die Methoden der Sozialwissenschaften: Zum 90’. Geburtstag von Sir Karl Popper. Wissenschaftspolitische Blätter.

[CR64] Milford, K. (2001 (1994)). In pursuit of rationality: A note of Hayek’s ‘The Counter-Revolution of Science’. In J. Birner & R. van Zijp (Eds.), *Hayek, co*-*ordination and evolution. His legacy in philosophy, politics, economics and the history of ideas* (pp. 323–340). London, New York: Routledge.

[CR65] Milford K, Cerman M, Berger P, Eigner P, Resch A (2011). Scheinsatzpositionen als Begründungsversuche der theoretischen Ökonomie: Schumpeters, Das Wesen und der Hauptinhalt der theoretischen Nationalökonomie‘. Die vielen Gesichter des wirtschaftlichen Wandels: Beiträge zur Innovationsgeschichte.

[CR66] Milonakis D, Fine B (2009). From political economy to economics: Method, the social and the historical in the evolution of economic theory. Economics as social theory.

[CR67] Mises L (1940). Nationalökonomie: Theorie des Handelns und Wirtschaftens.

[CR68] Mises, L. (1956). *Letter to Ludwig Lachmann*, 11.10.1956, Archive of the Mises Institute, Auburn, AL.

[CR69] Mises, R. (1968). *Positivism: A study in human understanding*. Unabridged republ. Dover Books on Philosophy. New York: Dover Publ.

[CR70] Mises, L. (1990 (1942)). Social science and natural science. In: R. Ebeling (Ed.), *Money, method, and the market process: Essays by Ludwig von Mises* (pp. 3–15). Auburn: Praxeology Press of the Ludwig von Mises Institute.

[CR71] Mises, L. (1990 (1944)). The treatment of ‘Irrationality’ in the social sciences. In R. Ebeling (Ed.), *Money, method, and the market process: Essays by Ludwig von Mises* (pp. 16–36). Auburn: Praxeology Press of the Ludwig von Mises Institute.

[CR72] Mises, L. (1998 (1949)). *Human action: A treatise on economics. The Scholar’s Edition.* Auburn: Mises Institute.

[CR73] Mises, L. (2003 (1933)). *Epistemological problems of economics*. Auburn: Mises Institute.

[CR74] Mises, L. (2005 (1957)). *Theory and history: An interpretation of social and economic evolution.* Liberty Fund Library of the Works of Ludwig von Mises. Indianapolis: Liberty Fund.

[CR75] Mises, L. (2009 (1940, 1978)). *Memoirs*. Auburn: Mises Institute.

[CR76] Mises, L. (2012 (1962)). *The ultimate foundation of economic science: An essay on method.* Princeton: Martino Fine Books.

[CR77] Nelson A, de Marchi N (1992). Reply by Alan Nelson. Post-Popperian methodology of economics: Recovering practice.

[CR78] Nozick R (1977). On Austrian methodology. Synthese.

[CR79] Oakley A (1997). Epistemological problems of human agency in Mises’s subjectivism. History of Economics Review.

[CR80] Oakley A (2000). Alfred Schütz and economics as social science. Human Studies.

[CR81] Oakley A (2002). Reconstructing economic theory. The problem of human agency.

[CR82] Oliva Cordoba M (2017). Uneasiness and scarcity: An analytic approach towards Ludwig von Mises’s Praxeology. Axiomathes.

[CR83] Otter N, Pies I, Leschke M (2010). Von der Praxeologie zur Rhetorik—zur Leistungsfähigkeit von zwei methodologischen Konzeptionen in der Ökonomie. Ludwig von Mises ökonomische Kommunikationswissenschaft.

[CR84] Pavlik, J. (2006). Austrian economics and the problem of apriorism. *E*-*Logios: Electronic Journal for Philosophy*. ISSN 1211-0442.

[CR85] Pham A (2017). Mainstream economics and the Austrian School: Toward reunification. Erasmus Journal for Philosophy and Economics.

[CR86] Poincaré, H. (1982 (1905, 1913)). *The foundations of science: Science and hypothesis, the value of science, science and method.* Lanham: University Press of America.

[CR87] Popper K (1975). Die offene Gesellschaft und ihre Feinde 1: Der Zauber Platons.

[CR88] Popper, K. (1980). *Die offene Gesellschaft und ihre Feinde 2: Falsche Propheten.* 6. Aufl. Uni-Taschenbücher 473. München: Francke.

[CR89] Popper, K. (2010 (1930–1933)). *Die beiden Grundprobleme der Erkenntnistheorie, 3. Aufl.,* Tübingen: Mohr Siebeck.

[CR90] Prychitko D, Boettke P (1998). Praxeology. The Elgar companion to Austrian economics.

[CR91] Psillos S (2011). Choosing the realist framework. Synthese.

[CR92] Puster R, Mises L (2014). Dualismen und ihre Hintergründe. Theorie und Geschichte: Eine Interpretation sozialer und wirtschaftlicher Entwicklung.

[CR93] Quine WVO (1951). Two dogmas of empiricism. Philosophical Review.

[CR94] Radnitzky G, Radnitzky G, Bouillon H (1995). Reply to Hoppe—On apriorism in Austrian economics. Values and society: Values and the social order.

[CR95] Rizzo M, Spadaro LM (1978). Praxeology and econometrics: A critique of positivist economics. New directions in Austrian economics.

[CR96] Rizzo M, Kirzner I (1982). Mises and Lakatos: A reformulation of Austrian methodology. Method, process, and Austrian economics: Essays in honor of Ludwig von Mises.

[CR97] Robbins L (1932). An essay on the nature and significance of economic science.

[CR98] Rothbard, M. (1997 (1956)). Toward a reconstruction of utility and welfare economics. In *The logic of action, volume one. Method, money and the Austrian School* (pp. 211–54). Cheltenham: Elgar.

[CR99] Rothbard, M. (1997 (1957)). In defense of “extreme apriorism”. In *The logic of action, volume one. Method, money and the Austrian School* (pp. 100–108). Cheltenham: Elgar.

[CR100] Rothbard, M. (1997 (1960)). The mantle of science. In *The logic of action, volume one. Method, money and the Austrian School* (pp. 3–23). Cheltenham: Elgar.

[CR101] Rothbard, M. (1997 (1973)). Praxeology as the method of the social sciences. In *The logic of action, volume one. Method, money and the Austrian School* (pp. 28–57). Cheltenham: Elgar.

[CR102] Rothbard, M. (1997 (1976)). Praxeology: The methodology of Austrian economics. In *The logic of action, volume one. Method, money and the Austrian School* (pp. 58–77). Cheltenham: Elgar.

[CR103] Rothbard M (1998). The ethics of liberty.

[CR104] Rothbard, M. (2007 (1985)). Preface. In L. Mises (Ed.), *Theory and history* (pp. xi–xix). Auburn: Mises Institute.

[CR105] Russell G (2008). Truth in virtue of meaning: A defence of the analytic/synthetic distinction.

[CR106] Samuelson P (1964). Theory and realism: A reply. The American Economic Review.

[CR107] Scheall S (2015). Hayek the Apriorist?. Journal of the History of Economic Thought.

[CR108] Scheall S (2015). A Hayekian explanation of Hayek’s ‘Epistemic Turn’. Economic Thought.

[CR109] Scheall, S. (2015c). Kinds of scientific rationalism: The case for methodological liberalism. *The Center for the History of Political Economy Working Paper Series*, 2015-05. Available at SSRN: https://ssrn.com/abstract=2619311.

[CR110] Scheall S (2017). What is so extreme about Mises’s extreme apriorism?. Journal of Economic Methodology.

[CR111] Scheall S (2017). Review of Alexander Linsbichler’s was Ludwig von Mises a conventionalist? A new analysis of the epistemology of the Austrian School of economics. Erasmus Journal for Philosophy and Economics.

[CR112] Schmid M, Pies I, Leschke M (2010). Ludwig von Mises’ Praxeologie. Ludwig von Mises‘ökonomische Argumentationswissenschaft.

[CR113] Schröder G, Pies I, Leschke M (2010). Kritik der unreinen Ökonomik: Ludwig von Mises’ Praxeologie als empiriefreier Ansatz einer logischen Ökonomik menschlicher Handlungen. Ludwig von Mises‘ökonomische Argumentationswissenschaft.

[CR114] Schütz, A. (1967 (1932)). *The phenomenology of the social world*. Evanston: Northwestern University Press.

[CR115] Selgin GA (1990). Praxeology and understanding: An analysis of the controversy in Austrian economics.

[CR116] Sidelle A (1989). Necessity, essence, and individuation: A defense of conventionalism.

[CR117] Smith B (1996). In defense of extreme (fallibilistic) apriorism. Journal of Libertarian Studies.

[CR118] Talmon JL (1985). Political messianism: The romantic phase.

[CR119] Talmon JL (1985). The origins of Totalitarian democracy.

[CR120] Tokumaru N, Parusniková Z, Cohen RS (2009). Popper’s analysis of the problem of induction and demarcation and Mises’ Justification of the theoretical social sciences. Rethinking Popper.

[CR121] White, L. (2003 (1977)). *The methodology of the Austrian School of economics*. Auburn: Mises Institute.

[CR122] Wieser F (1884). Über den Ursprung und die Hauptgesetze des wirthschaftlichen Wertes.

[CR123] Wright GH (1957). The logical problem of induction.

[CR124] Zanotti G, Cachanosky N (2015). Implications of Machlup’s interpretation of Mises’s epistemology. Journal of the History of Economic Thought.

[CR125] Zanotti, G., & Cachanosky, N. (2017). *What is so extreme about Mises’s extreme apriorism? Reply to Scott Scheall*. Available at SSRN: https://ssrn.com/abstract=2875122.

[CR126] Zilian H (1990). Klarheit und Methode: Felix Kaufmanns Wissenschaftstheorie.

